# Supplementary material for: Effects of realistic e-learning cases on students’ learning motivation during COVID-19
Source: PLoS One. 2021 Apr 21;16(4):e0249425. doi: 10.1371/journal.pone.0249425 (PMC8059845; doi:10.1371/journal.pone.0249425)
Supplement: S1 Text — (DOCX) [file pone.0249425.s001.docx]

**S1 Text - Supplementary Methods**

**Setting**

Due to the Covid-19 pandemic, the internal medicine curriculum at Heidelberg University Medical School (Heidelberg Curriculum Medicinale or HeiCuMed) was modified into e-learning-based teaching for summer semester 2020. The semester´s core structure of leading symptom-based teaching (Fig. 1) was adopted from its previous form and lectures and seminars were recorded and asynchronously made available for students. The semester normally consists of interdisciplinary leading-symptom based lectures and lectures in 5 modules for internal medicine subspecialties (cardiology/respiratory medicine/vascular medicine, hematology and oncology, gastroenterology, endocrinology, nephrology and general and psychosomatic medicine). Problem-based learning sessions were shifted to student-led online discussions. Skills labs contents (Nikendei et al. 2005) were available as commented learning videos known as Heidelberg Standard Procedures and Heidelberg Standard Physical Examination (Nikendei et al. 2016, Knauber et al. 2018), and Heidelberg Standard Communication.

**Case design**

E-learning cases were designed with Articulate (www.articulate.com). For each symptom-based teaching week (S1 Table), a clinical case was presented to the students and various quiz and interaction modes were included. Multiple audiovisual inputs were integrated e.g. prerecorded actual patient histories, recorded heart and lung sounds, pictures of histopathological findings and original videos of sonography, echocardiography and interventions (e.g. heart catheterization and endoscopy). Furthermore, students had to comment on presented reports from various clinical examinations; take decisions, select therapies and prescribe medications; and work through simulated conversations with patients, relatives and attendings. In addition, each case consisted of game-like interactions that required the student to apply practical and diagnostic skills to proceed with the patient e.g. performing a virtual clinical examination, analyzing a urine-specimen and perform echocardiogram and evaluating the consequent findings. For detailed information see S2 Table. Exemplary commented videos of 2 representative cases can be found as S1 Video and S2 Video.

S1 Video: <https://player.vimeo.com/video/492146693>

S2 Video: <https://player.vimeo.com/video/492146760>

The cases were designed in a manner that integration of recorded seminars and lectures and self-study was needed to answer the questions in a simulated clinical setting. Students were put into scenarios from emergency medicine, in- and off-patient setting and rehabilitation by working through an individual patient case. Performing one case every week in the 10 weeks internal medicine curriculum in a time-frame from Friday 12am (S1 Table) till next Monday was mandatory for students to be able to register for the final internal medicine exam. Performance was not rated by marks, but working through was automatically tracked by the learning management system “Moodle”.

**Case Shared Links**

Two of the cases (chest pain and edema) were translated into English from the original German version. Examples of translated cases can be found under the respective links.

**S1 Share Link** Case 1 – Chest Pain [Engl. version] <https://360.articulate.com/review/content/b8688dac-bbc5-485a-a6c9-6abc7a25fe26/review>

**S2 Share Link** Case 6 - Edema [Engl. version] <https://360.articulate.com/review/content/b8eb9cf0-943b-4081-8570-2397e7a93f5d/review>

**Case designs**

The online programming platform of Articulate was used for designing cases and their presentation. The Articulate software package consists of different sub-programs. On the one hand, the team used Articulate-Rise to create each case’s backbone and surface, and on the other hand, the game-like interactions were created in Articulate-Storyline 360 (Version Build 3.37.21614.0).

The cases were placed via a link directly into Heidelberg University Medical School’s e-learning platform Moodle (https://elearning-med.uni-heidelberg.de/).
