# Supplementary material for: Effects of realistic e-learning cases on students’ learning motivation during COVID-19
Source: PLoS One. 2021 Apr 21;16(4):e0249425. doi: 10.1371/journal.pone.0249425 (PMC8059845; doi:10.1371/journal.pone.0249425)
Supplement: S2 Text — (DOCX) [file pone.0249425.s002.docx]

**S2 Text - Original Survey Monkey Questionnaires [in German]**

**Fall 1: Thoraxschmerz**

Q1: Wie ansprechend findest Du das Design, die Aufmachung und der Aufbau? (Global Rating Scale)

Q2: Hat die Navigation im Fall gut funktioniert? (Global Rating Scale)

Q3: Hat die Bearbeitung und Beantwortung der verschiedenen Quizmodi

gut funktioniert? (Global Rating Scale)

Q4: Ist der Fall ein guter Abschluss für die LSV Woche? (Global Rating Scale)

Q5: Sind die Oberarztkommentare und Zitate hilfreich und informativ? (Global Rating Scale)

Q6: Hat Dich der Fall gut gefordert? (Global Rating Scale)

Q7: Was hat Dir besonders gut am Fall gefallen? (offene Freitextfrage)

Q8: Was können wir noch besser machen? (offene Freitextfrage)

Q9: Sind Dir Fehler aufgefallen? (offene Freitextfrage)

Q10: Möchtest du uns sonst noch etwas mitteilen? Insbesondere würde

uns bei dem Fall interessieren, ob das EKG-Tool bei Dir funktioniert hat. (offene Freitextfrage)

**Fall 2: Bauchschmerz**

Q1: Wie findest du den Fall insgesamt? (Global Rating Scale)

Q2: Hat die Navigation im Fall gut funktioniert? (Global Rating Scale)

Q3: Hat die Bearbeitung und Beantwortung der verschiedenen Quizmodi

gut funktioniert? (Global Rating Scale)

Q4: Ist der Fall ein guter Abschluss für die LSV Woche? (Global Rating Scale)

Q5: Sind die Oberarztkommentare und Zitate hilfreich und informativ? (Global Rating Scale)

Q6: Hat Dich der Fall gut gefordert? (Global Rating Scale)

Q7: Was hat Dir besonders gut am Fall gefallen? (offene Freitextfrage)

Q8: Was können wir noch besser machen? Sind Dir Fehler aufgefallen? (offene Freitextfrage)

Q9: Welche interaktiven Elemente / Inhalte würdest Du Dir für weitere Fälle zu LSV Bauchschmerz und LSV Thoraxschmerz wünschen? (offene Freitextfrage)

Q10: Hättest Du auch in anderen klinischen Fächern gerne E-Learning-

Fälle unseres Formats zur Verfügung bzw. wie findest Du das Format? (offene Freitextfrage)

**Fall 3: Fieber**

Q1: Wie findest du den Fall insgesamt? (Global Rating Scale)

Q2: Hat die Navigation im Fall gut funktioniert? (Global Rating Scale)

Q3: Hat die Bearbeitung und Beantwortung der verschiedenen Quizmodi

gut funktioniert? (Global Rating Scale)

Q4: Ist der Fall ein guter Abschluss für die LSV Woche? (Global Rating Scale)

Q5: Sind die Oberarztkommentare und Zitate hilfreich und informativ? (Global Rating Scale)

Q6: Hat Dich der Fall gut gefordert? (Global Rating Scale)

Q7: Was hat Dir besonders gut am Fall gefallen? (offene Freitextfrage)

Q8: Was können wir noch besser machen? Sind Dir Fehler aufgefallen? (offene Freitextfrage)

Q9: Welche interaktiven Elemente / Inhalte würdest Du Dir für weitere

Fälle zur LSV Fieber wünschen? (offene Freitextfrage)

Q10: Welche Lerninhalte wünscht Du Dir neben den LSV´s noch gerne

zusätzlich als E-Learning? (offene Freitextfrage)

**Fall 4: Luftnot**

Q1: Wie findest du den Fall insgesamt? (Global Rating Scale)

Q2: Hat Dir die Bearbeitung des Falls Spaß gemacht? (Global Rating Scale)

Q3: Hat die Bearbeitung und Beantwortung der verschiedenen Quizmodi

gut funktioniert? (Global Rating Scale)

Q4: Ist der Fall ein guter Abschluss für die LSV Woche? (Global Rating Scale)

Q5: Sind die Oberarztkommentare und Zitate hilfreich und informativ? (Global Rating Scale)

Q6: Hat Dich der Fall gut gefordert? (Global Rating Scale)

Q7: Was hat Dir besonders gut am Fall gefallen? (offene Freitextfrage)

Q8: Was können wir noch besser machen? Sind dir Fehler aufgefallen

und welches Gerät benutzt Du zur Bearbeitung (Handy/Tablet/Computer

- Apple/Microsoft)? (offene Freitextfrage)

Q9: Welche interaktiven Elemente / Inhalte würdest Du Dir für weitere

Fälle zur LSV Luftnot wünschen? (offene Freitextfrage)

Q10: Wie findest Du es, dass die Fälle verpflichtend sind? (offene Freitextfrage)

**Fall 5: Müdigkeit**

Q1: Wie findest du den Fall insgesamt? (Global Rating Scale)

Q2: Hat die Bearbeitung und Beantwortung der verschiedenen Quizmodi

gut funktioniert? (Global Rating Scale)

Q3: Ist der Fall ein guter Abschluss für die LSV Woche? (Global Rating Scale)

Q4: Sind die Oberarztkommentare und Zitate hilfreich und informativ? (Global Rating Scale)

Q5: Hat Dich der Fall gut gefordert? (Global Rating Scale)

Q6: Wie lange sollte deiner Meinung nach die Bearbeitung eines Falls

dauern? (Global Rating Scale)

Q7: Was hat Dir besonders gut am Fall gefallen? (offene Freitextfrage)

Q8: Was können wir noch besser machen? Sind dir Fehler aufgefallen? (offene Freitextfrage)

Q9: Welche interaktiven Elemente / Inhalte würdest Du Dir für weitere

Fälle zur LSV Müdigkeit wünschen? (offene Freitextfrage)

Q10: Welche interaktiven Elemente wünscht Du Dir generell in allen

Fällen? (offene Freitextfrage)

**Fall 6: Wassereinlagerung**

Q1: Wie findest du den Fall insgesamt? (Global Rating Scale)

Q2: Hat Dir die Bearbeitung des Falls Spaß gemacht? (Global Rating Scale)

Q3: Hat die Bearbeitung und Beantwortung der verschiedenen Quizmodi

gut funktioniert? (Global Rating Scale)

Q4: Ist der Fall ein guter Abschluss für die LSV Woche? (Global Rating Scale)

Q5: Sind die Oberarztkommentare und Zitate hilfreich und informativ? (Global Rating Scale)

Q6: Hat Dich der Fall gut gefordert? (Global Rating Scale)

Q7: Was hat Dir besonders gut am Fall gefallen? (offene Freitextfrage)

Q8: Was können wir noch besser machen? Sind dir Fehler aufgefallen

und welches Gerät benutzt Du zur Bearbeitung (Handy/Tablet/Computer

- Apple/Microsoft)? (offene Freitextfrage)

Q9: Welche interaktiven Elemente / Inhalte würdest Du Dir für weitere

Fälle zur LSV Wassereinlagerung wünschen? (offene Freitextfrage)

Q10: Die oder das Nutella? (offene Freitextfrage)

**Fall 7: Gelbsucht**

Q1: Wie findest du den Fall insgesamt? (Global Rating Scale)

Q2: Hat Dir die Bearbeitung des Falls Spaß gemacht? (Global Rating Scale)

Q3: Hat die Bearbeitung und Beantwortung der verschiedenen Quizmodi

gut funktioniert? (Global Rating Scale)

Q4: Ist der Fall ein guter Abschluss für die LSV Woche? (Global Rating Scale)

Q5: Sind die Oberarztkommentare und Zitate hilfreich und informativ? (Global Rating Scale)

Q6: Hat Dich der Fall gut gefordert? (Global Rating Scale)

Q7: Was hat Dir besonders gut am Fall gefallen? (offene Freitextfrage)

Q8: Was können wir noch besser machen? Sind dir Fehler aufgefallen? (offene Freitextfrage)

Q9: Welche interaktiven Elemente / Inhalte würdest Du Dir für weitere

Fälle zur LSV Gelbsucht wünschen? (offene Freitextfrage)

Q10: Hast Du Dich nach der Bearbeitung der Fälle nochmal mehr mit einer Thematik beschäftigt? (offene Freitextfrage)

**Fall 8: Schwindel**

Q1: Wie findest du den Fall insgesamt? (Global Rating Scale)

Q2: Hat Dir die Bearbeitung des Falls Spaß gemacht? (Global Rating Scale)

Q3: Hat die Bearbeitung und Beantwortung der verschiedenen Quizmodi

gut funktioniert? (Global Rating Scale)

Q4: Ist der Fall ein guter Abschluss für die LSV Woche? (Global Rating Scale)

Q5: Sind die Oberarztkommentare und Zitate hilfreich und informativ? (Global Rating Scale)

Q6: Hat Dich der Fall gut gefordert? (Global Rating Scale)

Q7: Was hat Dir besonders gut am Fall gefallen? (offene Freitextfrage)

Q8: Was können wir noch besser machen? Sind dir Fehler aufgefallen? (offene Freitextfrage)

Q9: Welche interaktiven Elemente / Inhalte würdest Du Dir für weitere

Fälle zur LSV Schwindel, Synkope, Bewusstlosigkeit wünschen? (offene Freitextfrage)

**Fall 9: Gewichtsverlust**

Q1: Wie findest du den Fall insgesamt? (Global Rating Scale)

Q2: Hat Dir die Bearbeitung des Falls Spaß gemacht? (Global Rating Scale)

Q3: Hat die Bearbeitung und Beantwortung der verschiedenen Quizmodi

gut funktioniert? (Global Rating Scale)

Q4: Ist der Fall ein guter Abschluss für die LSV Woche? (Global Rating Scale)

Q5: Sind die Oberarztkommentare und Zitate hilfreich und informativ? (Global Rating Scale)

Q6: Hat Dich der Fall gut gefordert? (Global Rating Scale)

Q7: Was hat Dir besonders gut am Fall gefallen? (offene Freitextfrage)

Q8: Was können wir noch besser machen? Sind dir Fehler aufgefallen? (offene Freitextfrage)

Q9: Welche interaktiven Elemente / Inhalte würdest Du Dir für weitere

Fälle zur LSV Gewichtsverlust wünschen? (offene Freitextfrage)

**Fall 10: Schmerzen des Bewegungsapparats**

Q1: Wie findest du den Fall insgesamt? (Global Rating Scale)

Q2: Hat Dir die Bearbeitung des Falls Spaß gemacht? (Global Rating Scale)

Q3: Hat die Bearbeitung und Beantwortung der verschiedenen Quizmodi

gut funktioniert? (Global Rating Scale)

Q4: Sind die Oberarztkommentare und Zitate hilfreich und informativ? (Global Rating Scale)

Q5: Hat Dich der Fall gut gefordert? (Global Rating Scale)

Q6: Was hat Dir besonders gut am Fall gefallen? (offene Freitextfrage)

Q7: Was können wir noch besser machen? Sind dir Fehler aufgefallen? (offene Freitextfrage)

Q8: Welche interaktiven Elemente / Inhalte würdest Du Dir für weitere

Fälle zur LSV Schmerzen des Bewegungsapparats wünschen? (offene Freitextfrage)

Q9: Hättest Du lieber 3 kurze oder einen langen Fall in jeder LSV-Woche? (offene Freitextfrage)

Q10: Wie hat Dir das Quiz nach dem Fall gefallen? (offene Freitextfrage)

**Abschlussevaluation**

Q1-Q15 (Global Rating Scale) Q16-Q20 (offene Freitextfrage)

Q1: Wie sehr hast Du dich auf die Fallbearbeitung gefreut?

Q2: Wie gut war der Fall Thoraxschmerz für Dich als Lernüberprüfung?

Q3: Wie gut war der Fall Bauchschmerz für Dich als Lernüberprüfung?

Q4: Wie gut war der Fall Fieber für Dich als Lernüberprüfung?

Q5: Wie gut war der Fall Luftnot für Dich als Lernüberprüfung?

Q6: Wie gut war der Fall Müdigkeit für Dich als Lernüberprüfung?

Q7: Wie gut war der Fall Wassereinlagerung für Dich als Lernüberprüfung?

Q8: Wie gut war der Fall Gelbsucht für Dich als Lernüberprüfung?

Q9: Wie gut war der Fall Schwindel, Synkope, Bewusstlosigkeit für Dich als Lernüberprüfung?

Q10: Wie gut war der Fall Gewichtsverlust für Dich als Lernüberprüfung?

Q11: Wie gut war der Fall Schmerzen des Bewegungsapparats für Dich als Lernüberprüfung?

Q12: Wie viel Zeit hast du mit der Bearbeitung eines Falls verbracht?

Q13: Hast du Fragen, die sich aus der Bearbeitung der Fälle ergeben haben, recherchiert? Ja oder Nein?

Q14: Wie viel Zeit hast Du mit der weiterführenden Recherche pro Fall verbracht?

Q15: Wie gut waren die Fälle, um Dein Verständnis für klinische Prozesse und Abläufe zu verbessern?

Q16: Wirst Du die Fälle nochmals zur Klausurvorbereitung nutzen?

Q17: Welchen Mehrwert haben die Fälle für Dich inhaltlich gebracht?

Q18: Wie hat die Lernatmosphäre im klinischen Setting Deine Ambition während der Bearbeitung verglichen mit der Bearbeitung von MC Fragen beeinflusst?

Q19: Welchen Einfluss hatten die Fälle auf Deine Lernstruktur und Dein Lernverhalten?

Q20: Welchen Einfluss hatten die Fälle auf Deine Lernmotivation?
