# Supplementary material for: Effects of realistic e-learning cases on students’ learning motivation during COVID-19
Source: PLoS One. 2021 Apr 21;16(4):e0249425. doi: 10.1371/journal.pone.0249425 (PMC8059845; doi:10.1371/journal.pone.0249425)
Supplement: S3 Text — (DOCX) [file pone.0249425.s003.docx]

**S3 Text - Original Survey Monkey Questionnaires [translated to English]**

**Case 1: Chest pain**

Q1: How appealing do you find the design, the presentation and the structure of the case? (Global Rating Scale)

Q2: Did the navigation work well in the case? (Global Rating Scale)

Q3: Did the editing and answering of the different quiz modes work well? (Global Rating Scale)

Q4: Is the case a good ending for the LSV week? (Global Rating Scale)

Q5: Are the senior physician comments and quotes helpful and informative? (Global Rating Scale)

Q6: Did the case challenge you well? (Global Rating Scale)

Q7: What did you particularly like about the case? (Free text question)

Q8: What can we do even better? (Free text question)

Q9: Have you noticed any errors? (Free text question)

Q10: Anything else you'd like to share? In particular, we would be interested to know whether the ECG tool worked for you. (Free text question)

**Case 2: Abdominal pain**

Q1: What do you think of the case overall? (Global Rating Scale)

Q2: Did the navigation work well in the case? (Global Rating Scale)

Q3: Did the editing and answering of the different quiz modes work well? (Global Rating Scale)

Q4: Is the case a good ending for the LSV week? (Global Rating Scale)

Q5: Are the senior physician comments and quotes helpful and informative? (Global Rating Scale)

Q6: Did the case challenge you well? (Global Rating Scale)

Q7: What did you particularly like about the case? (Free text question)

Q8: What can we do better? Have you noticed any errors? (Free text question)

Q9: What interactive elements / content would you like to see for more cases on LSV Abdominal Pain and LSV Thoracic Pain? (Free text question)

Q10: Would you like to have e-learning cases of this format available in other clinical subjects and what do you think of the format? (Free text question)

**Case 3: Fever**

Q1: What do you think of the case overall? (Global Rating Scale)

Q2: Did the navigation work well in the case? (Global Rating Scale)

Q3: Did the editing and answering of the different quiz modes work well? (Global Rating Scale)

Q4: Is the case a good ending for the LSV week? (Global Rating Scale)

Q5: Are the senior physician comments and quotes helpful and informative? (Global Rating Scale)

Q6: Did the case challenge you well? (Global Rating Scale)

Q7: What did you particularly like about the case? (Free text question)

Q8: What can we do better? Have you noticed any errors? (Free text question)

Q9: What interactive elements / content would you like to see in more cases on LSV Fever? (Free text question)

Q10: What additional learning content would you like to see as e-learning in addition to the LSVs? (Free text question)

**Case 4: Dyspnea**

Q1: What do you think of the case overall? (Global Rating Scale)

Q2: Did working through the cases bring you fun? (Global Rating Scale)

Q3: Did the editing and answering of the different quiz modes work well? (Global Rating Scale)

Q4: Is the case a good ending for the LSV week? (Global Rating Scale)

Q5: Are the senior physician comments and quotes helpful and informative? (Global Rating Scale)

Q6: Did the case challenge you well? (Global Rating Scale)

Q7: What did you particularly like about the case? (Free text question)

Q8: What can we do better? Did you notice any errors and which device do you use for editing (mobile phone/tablet/computer - Apple/Microsoft)? (Free text question)

Q9: What interactive elements / content would you like to see for more cases on LSV air distress? (Free text question)

Q10: How do you feel about cases being mandatory? (Free text question)

**Case 5: Fatigue**

Q1: What do you think of the case overall? (Global Rating Scale)

Q2: Did the editing and answering of the different quiz modes work well? (Global Rating Scale)

Q3: Is the case a good ending for the LSV week? (Global Rating Scale)

Q4: Are the senior physician comments and quotes helpful and informative? (Global Rating Scale)

Q5: Did the case challenge you well? (Global Rating Scale)

Q6: How long do you think it should take to process a case? (Global Rating Scale)

Q7: What did you particularly like about the case? (Free text question)

Q8: What can we do better? Have you noticed any errors? (Free text question)

Q9: What interactive elements / content would you like to see in more cases on LSV fatigue? (Free text question)

Q10: What interactive elements would you generally like to see in all cases? (Free text question)

**Case 6: Edema**

Q1: What do you think of the case overall? (Global Rating Scale)

Q2: Did you enjoy working on the case? (Global Rating Scale)

Q3: Did the editing and answering of the different quiz modes work well? (Global Rating Scale)

Q4: Is the case a good ending for the LSV week? (Global Rating Scale)

Q5: Are the senior physician comments and quotes helpful and informative? (Global Rating Scale)

Q6: Did the case challenge you well? (Global Rating Scale)

Q7: What did you particularly like about the case? (Free text question)

Q8: What can we do better? Did you notice any errors and which device do you use for editing (mobile phone/tablet/computer - Apple/Microsoft)? (Free text question)

Q9: What interactive elements / content would you like to see for more cases on LSV water retention? (Free text question)

Q10: The (Die) Nutella or the (Das) Nutella? (Free text question)

**Case 7: Jaundice**

Q1: What do you think of the case overall? (Global Rating Scale)

Q2: Did you enjoy working on the case? (Global Rating Scale)

Q3: Did the editing and answering of the different quiz modes work well? (Global Rating Scale)

Q4: Is the case a good ending for the LSV week? (Global Rating Scale)

Q5: Are the senior physician comments and quotes helpful and informative? (Global Rating Scale)

Q6: Did the case challenge you well? (Global Rating Scale)

Q7: What did you particularly like about the case? (Free text question)

Q8: What can we do better? Have you noticed any errors? (Free text question)

Q9: What interactive elements/content would you like to see for other cases on LSV jaundice? (Free text question)

Q10: After working on the cases, did you look into the subject matter more? (Free text question)

**Case 8: Dizziness**

Q1: What do you think of the case overall? (Global Rating Scale)

Q2: Did you enjoy working on the case? (Global Rating Scale)

Q3: Did the editing and answering of the different quiz modes work well? (Global Rating Scale)

Q4: Is the case a good ending for the LSV week? (Global Rating Scale)

Q5: Are the senior physician comments and quotes helpful and informative? (Global Rating Scale)

Q6: Did the case challenge you well? (Global Rating Scale)

Q7: What did you particularly like about the case? (Free text question)

Q8: What can we do better? Have you noticed any errors? (Free text question)

Q9: What interactive elements / content would you like to see for more cases on LSV dizziness, syncope, unconsciousness? (Free text question)

**Case 9: Weight loss**

Q1: What do you think of the case overall? (Global Rating Scale)

Q2: Did you enjoy working on the case? (Global Rating Scale)

Q3: Did the editing and answering of the different quiz modes work well? (Global Rating Scale)

Q4: Is the case a good ending for the LSV week? (Global Rating Scale)

Q5: Are the senior physician comments and quotes helpful and informative? (Global Rating Scale)

Q6: Did the case challenge you well? (Global Rating Scale)

Q7: What did you particularly like about the case? (Free text question)

Q8: What can we do better? Have you noticed any errors? (Free text question)

Q9: What interactive elements / content would you like to see in more LSV weight loss cases? (Free text question)

**Case 10 Musculoskeletal pain**

Q1: What do you think of the case overall? (Global Rating Scale)

Q2: Did you enjoy working on the case? (Global Rating Scale)

Q3: Did the editing and answering of the different quiz modes work well? (Global Rating Scale)

Q4: Are the senior physician comments and quotes helpful and informative? (Global Rating Scale)

Q5: Did the case challenge you well? (Global Rating Scale)

Q6: What did you particularly like about the case? (Free text question)

Q7: What can we do better? Have you noticed any errors? (Free text question)

Q8: What interactive elements / content would you like to see in more cases on LSV musculoskeletal pain? (Free text question)

Q9: Would you rather have 3 short cases or one long case in each LSV week? (free text question)

Q10: How did you like the quiz after the case? (Free text question)

**Final evaluation**

Q1-Q15 (Global Rating Scale) Q16-Q20 (Free text question)

Q1: How much were you looking forward to the casework?

Q2: How good was the thoracic pain case for you as a learning review?

Q3: How good was the abdominal pain case for you as a learning review?

Q4: How good was the fever case for you as a learning review?

Q5: How good was the air distress case for you as a learning review?

Q6: How good was the fatigue case for you as a learning review?

Q7: How good was the water retention case for you as a learning review?

Q8: How good was the jaundice case for you as a learning review?

Q9: How good was the dizziness, syncope, unconsciousness case for you as a learning review?

Q10: How good was the weight loss case for you as a learning review?

Q11: How good was the musculoskeletal pain case for you as a learning review?

Q12: On average, how much time did you spend working on a case?

Q13: Did you research questions that arose from working on the cases? Yes or no?

Q14: How much time did you spend on advanced research per case?

Q15: How good were the cases in improving your understanding of clinical processes and procedures?

Q16: Will you use the cases again for exam preparation?

Q17: What added value did the cases bring for you in terms of content?

Q18: How did the learning atmosphere in the clinical setting affect your ambition during editing compared to editing MC questions?

Q19: What impact did the cases have on your learning structure and behavior?

Q20: What impact did the cases have on your motivation to learn?
