# Supplementary material for: Effects of realistic e-learning cases on students’ learning motivation during COVID-19
Source: PLoS One. 2021 Apr 21;16(4):e0249425. doi: 10.1371/journal.pone.0249425 (PMC8059845; doi:10.1371/journal.pone.0249425)
Supplement: S1 Table — (DOCX) [file pone.0249425.s004.docx]

**S1 Table Design of HeiCuMed Internal Medicine Curriculum**

|  | Monday | Thursday | Wednesday | Thursday | Friday |  |  |  |  |
| --- | --- | --- | --- | --- | --- | --- | --- | --- | --- |
| 08:30 | Interdisciplinary leading symptom-based  lecture **08:30-10:00** | PBL **08:30-10:00** | Interdisciplinary  leading symptom-based  lecture **08:30-10:00** | Interdisciplinary leading symptom-based  lecture **08:30-10:00** | Interdisciplinary leading symptom-based  lecture **08:30-10:00** |  |  |  |  |
| 09:00 |  |  |  |  |  |  | **Symptom-Based week schedule** | | |
| 09:30 |  |  |  |  |  |  | Week 1 | Chest pain |  |
| 10:00 |  |  |  |  |  |  | Week 2 | Abdominal pain | |
|  |  |  |  |  |  |  | Week 3 | Fever |  |
| 10:15 | Internal medicine subspecialties seminars  **10:15-12:15** | | | Rotation Skills Lab/ bed-side-teaching **10:15-11:45** | Internal medicine subspecialties seminars  **10:15-12:15** |  | Week 4 | Dyspnea |  |
| 11:00 |  |  |  |  |  |  | Week 5 | Fatigue |  |
| 11:30 |  |  |  |  |  |  | Week 6 | Edema |  |
| 12:00 |  |  |  |  |  |  | Week 7 | Icterus |  |
| 12:15 |  |  |  | Rotation Skills Lab/ bed-side-teaching **11:50-13:20** |  |  | Week 8 | Vertigo, syncope, LOC | |
| 12:30 | Self-study  advanced skills |  |  |  | Articulate  e-learning cases |  | Week 9 | Weight loss |  |
| 13:00 |  | General medicine lecture (10x) **13:00-14:00** | Rotation Skills  Lab/MediKit bed-side-teaching **12:45-14:15** |  |  |  | Week 10 | Musculoskeletal pain | |
| 13:30 | Oncology lecture **13:30-14:30** |  |  | Rotation Skills Lab/MediKit bed-side-teaching **13:30-15:00** | Free practice  training/self-study  **12:30-17:00** |  |  |  |  |
| 14:30 |  |  |  |  |  |  |  |  |  |
| 15:00 | Self-study  advanced skills lab from 12:30 |  | Rotation Skills  Lab/MediKit bed-side-teaching **14:30-16:00** |  |  |  |  |  |  |
| 15:30 |  |  |  |  |  |  |  |  |  |
| 16:00 |  |  |  |  |  |  |  |  |  |
| 16:30 |  |  | Rotation Skills Lab/ bed-side-teaching **16:15-17:45** |  |  |  |  |  |  |
| 17:00 | facultative  ECG-course 17:00-18:30 |  |  |  |  |  |  |  |  |
| 17:30 |  |  |  |  |  |  |  |  |  |
| 18:00 |  |  |  |  |  |  |  |  |  |
