# Supplementary material for: Effects of realistic e-learning cases on students’ learning motivation during COVID-19
Source: PLoS One. 2021 Apr 21;16(4):e0249425. doi: 10.1371/journal.pone.0249425 (PMC8059845; doi:10.1371/journal.pone.0249425)
Supplement: S2 Table — (DOCX) [file pone.0249425.s005.docx]

**S2 Table – Case Overview of embedded interactive tools**

|  | Spoken audio | Interactive tool | Quiz | Interactive conversation | Video | Audio | Original  findings | Other |
| --- | --- | --- | --- | --- | --- | --- | --- | --- |
| **Case 1**  **Thoracic pain** | Emergency handover  Spoken PCI findings | ECG | Troponin T Dynamic  ECG Drag and Drop  Heart catheterization | Conversation scenario with patient’s wife |  |  |  |  |
| **Case 2 Abdominal pain** | patient problems |  | Coloscopy finding  Laboratory quiz | Conversation scenario with patient for taking history  Interactive conversation scenario with patient’s mother | Coloscopy |  |  | WhatsApp Scenario |
| **Case 3**  **Fever** | History of patient |  | Blood smear pictures |  |  |  | Original Laboratory result  Original Pathology picture |  |
| **Case 4**  **Dyspnea** | History of patient | Interactive  emergency physical examination  Allergy testing | Quiz: lung function analysis | Interactive conversation scenario with patient’s teacher  Interactive conversation scenario with patient | Capillary blood taking instructions | Breathing sounds of patient | Emergency Monitoring  Blood gas analysis |  |
| **Case 5**  **Fatigue** | Emergency handover  Report of befriended doctor | Physical examination |  | Interactive conversation scenario with patient  Interactive conversation scenario with patient II |  |  | Vital parameters monitoring  Blood gas analysis  PHQ-9 Questionnaire |  |
| **Case 6**  **Water retention** |  | Interactive physical examination  Interactive UrinStix and following finding  Interactive Lesson about secondary complications of chronic kidney disease | Nephrotic versus Nephritic syndrome Drag and Drop  Pathology findings Drag and Drop with original findings  Urinary obstruction Drag and Drop with original findings  Interactive quiz: Diuretics and their place of action | Interactive educational talk with the patient |  |  | Letter of referral to a specialist |  |
| **Case 7**  **Jaundice** | Patient description | Interactive physical examination | Quiz: Different types of jaundice Drag and Drop  Quiz: various hepatitis  Quiz: Associate elevated liver values with different diseases | Conversation with the senior doctor  Interactive anamnesis discussion with the patient  Final discussion with the senior doctor |  |  | Liver ultrasound |  |
| **Case 8 Dizziness, syncope, loss of consciousness** | Voice Audio: Paramedic | Interactive physical examination | Quiz: Diagnosis of different auscultations  Quiz: ECG findings  Quiz: Echocardiography findings  Quiz: Therapeutic decision TAVI vs SAVR Drag and Drop |  | TAVI-Implan-tation |  | Echocardiography |  |
| **Case 9 Weight loss** | Spoken Sonographic findings |  | Laboratory quiz  Quiz: Recognize structures in thyroid ultrasound | Interactive consultation with a colleague  Educational talk with the patient |  |  | Thyroid scintigraphy |  |
| **Case 10 Musculoskeletal pain** | Patient report | Interactive physical examination | Quiz: Osteoarthritis and arthritis Drag and Drop  Quiz: Diagnosis of various X-ray findings of the hand  Laboratory quiz  Rheumatology quiz |  |  |  |  |  |
