# Supplementary figures and images for: Effects of realistic e-learning cases on students’ learning motivation during COVID-19
Source: PLoS One. 2021 Apr 21;16(4):e0249425. doi: 10.1371/journal.pone.0249425 (PMC8059845; doi:10.1371/journal.pone.0249425)

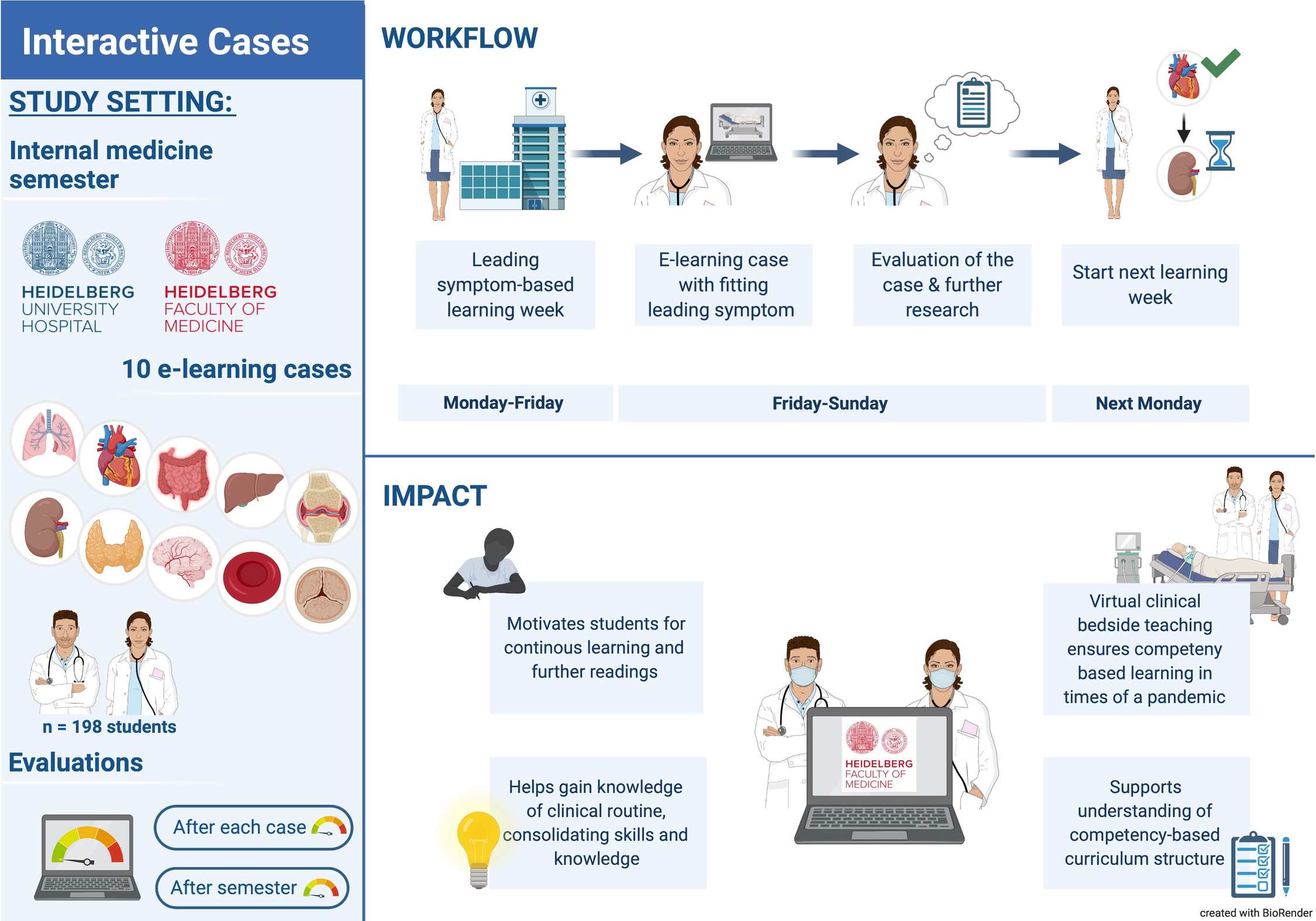

Supplement: S1 Graphical abstract — (TIF) [file pone.0249425.s010.tif]
